# Possible GABAkine‐Mediated Sedative‐Like Antidepressant Effects of Phytol: Molecular Interventions Through In Vitro, In Vivo and In Silico Approaches

**DOI:** 10.1111/cns.70350

**Published:** 2025-03-21

**Authors:** Md. Torequl Islam, Jannatul Ferdous, Md. Sakib Al Hasan, Md. Shimul Bhuia, Irfan Aamer Ansari, Siddique Akber Ansari, Md. Amirul Islam, Md. Saifuzzaman

**Affiliations:** ^1^ Pharmacy Discipline Khulna University Khulna Bangladesh; ^2^ Department of Pharmacy Gopalganj Science and Technology University Gopalganj Bangladesh; ^3^ Bioinformatics and Drug Innovation Laboratory BioLuster Research Center Ltd. Gopalganj Bangladesh; ^4^ Department of Biotechnology and Genetic Engineering Gopalganj Science and Technology University Gopalganj Bangladesh; ^5^ Microbial Biotechnology Division National Institute of Biotechnology Dhaka Bangladesh; ^6^ Department of Drug Science and Technology University of Turin Turin Italy; ^7^ Department of Pharmaceutical Chemistry College of Pharmacy, King Saud University Riyadh Saudi Arabia; ^8^ Department of Pharmacy East West University Dhaka Bangladesh

**Keywords:** antidepressant effect, GABAkine pathway, molecular docking, phytol

## Abstract

**Background:**

A previous report suggests that phytol (PHY) may exert its antidepressant effects in mice, possibly through GABA_A_ receptor interaction pathways.

**Aim:**

We aimed to check its antidepressant effect with possible molecular mechanisms through behavioral and *in silico* studies.

**Methods:**

For this, adult mice were randomly divided into different groups (*n* = 6), namely control (vehicle), standards (DZP: diazepam at 2 mg/kg, FLU: flumazenil at 0.1 mg/kg, FLUX: fluoxetine at 20 mg/kg), PHY (25, 50, and 75 mg/kg), and combined groups (PHY‐75 with DZP‐2 and/or FLU‐0.1, and FLUX‐20). Thirty minutes after treatment, each animal was subjected to tail suspension and forced swimming tests, and their immobility time (IMT) was counted for 5 min. In silico studies were performed with the GABA_A_ receptor α1, α2, α3, α5, and γ2 subunits and 5HT_1A_ to investigate possible molecular mechanisms. Additionally, in vitro GABA activity of PHY and/or reference drugs was also performed by using the colorimetric method.

**Results:**

The results demonstrated that PHY and/or DZP significantly (*p* < 0.05) and concentration‐dependently inhibited GABA, while FLU alone or its combination with PHY reversed it. In mice, PHY dose‐dependently reduced the IMT in both protocols, while FLUX‐20 showed lower IMT compared to the control and DZP, indicating elevated locomotion in mice. It showed a reduced IMT value in male animals than in female animals. In both sexes, PHY at 75 mg/kg significantly (*p* < 0.05) increased the IMT values with DZP‐2, while reducing this parameter with FLU‐0.1. *In silico* studies demonstrated that PHY exhibited higher binding affinities with the α2 and α3 subunits of the GABA_A_ and 5HT_1A_ receptors by −6.5, −7.2 and 6.7 kcal/mol, respectively.

**Conclusion:**

Taken together, PHY exerted sedative‐*like* antidepressant effects in mice and modulated the effects of GABAergic drugs DZP and FLU and serotonergic drug FLUX. PHY may be a potential candidate for the management of depression.

## Graphical abstract

Phytol (PHY) concentration‐dependently inhibited GABA activity and dose‐dependently reduced immobility time (IMT) in Swiss mice. It also modulated the GABA activity and IMT values of diazepam and flumazenil. It showed a slight sex‐dependent antidepressant effect on mice, possibly through the GABAA receptor interaction pathway.

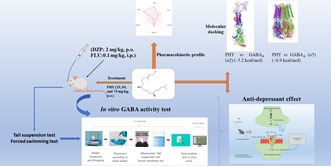

## 1 Introduction

Depression is considered the second‐leading psychiatric disorder in the world. Approximately 21% of people suffer from depression globally [1]. Previously, the suspected age range was 40–50 years, but at present, it falls within the age range between 25 and 35 years [2]. To date, some antidepressants have been introduced to manage depression, including tricyclic antidepressants, monoamine oxidase inhibitors [3], and selective serotonin reuptake inhibitors (SSRI). However, all these drugs have mild to serious side effects such as insomnia, anxiety, weight gain, and so on. Undoubtedly, nature has provided a wide window for the best and safest medication since the beginning of human life on the planet [4].

The incidence of depression is linked to the dysfunction of the gamma‐aminobutyrate (GABA) and serotonin reuptake systems [5, 6]. Many antidepressants act through this pathway. GABA exerts a broad effect on the nervous system by antagonizing the excitatory effects of glutamate, which is involved in many psychiatric diseases. Cumulative literature demonstrates that a low GABA level may result in depression in animals [7]. GABA receptors antagonism may initiate depression and depression‐like disorders in humans [8, 9]. It is evident that activation of the GABA_A_ receptors results in antidepressant‐like effects in experimental animals [10]. Therefore, targeting the neurotransmitter GABA or its functional receptors, GABA_A_, might be one of the potential approaches to treating depression and developing new GABAergic antidepressant drugs.

Different preclinical and clinical reports suggest that GABA is interconnected with depression [11]. Patients with severe depressive illness have lower amounts of GABA in their plasma and corticospinal fluid, and GABA levels improved after using selective serotonin uptake inhibitors [12, 13]. GABA_A_ receptors, including α2 and α5 receptors, have higher expression levels in limbic areas [14] that have a role in processing emotional stimuli and are connected to the pathogenesis of depression [15, 16]. Additionally, the amelioration of cognitive abnormalities in schizophrenia has been associated with the α2 subunit of GABA_A_ receptors [17]. Moreover, another subunit of GABA receptors, including α1 and α3, causes the antidepressant‐like effects of the benzodiazepine diazepam in mice [18].

In depression, presynaptic 5HT_1A_ autoreceptors, located on serotonin‐releasing neurons, play a vital role in regulating serotonin release into the synaptic cleft. These autoreceptors act as a feedback mechanism that inhibits further serotonin release when activated. In depressive states, increased sensitivity or overactivity of these autoreceptors reduces serotonin release, lowering its availability in the synapse and contributing to depressive symptoms. Antidepressants, like SSRIs, work by increasing serotonin levels over time, which reduces presynaptic inhibition, eventually desensitizing the autoreceptors and promoting sustained serotonin release to alleviate depression [19].

Certain drugs acting through the central nervous system (CNS), especially through GABA receptors, can exert both anxiolytic and sedative/antidepressant effects on animals; for example, benzodiazepine (BDZ) [20, 21]. Phytol (PHY: 3,7,11,15‐tetramethylhexadec‐2‐en‐1‐ol), a chlorophyll‐derived diterpenoid, possesses diverse biological activities, such as antioxidant, inflammatory and immune‐modulating, antimicrobial, anticancer, and organ protective, including neuroprotective effects [22, 23, 24]. Many studies report that PHY may exert its anxiolytic, anti‐depressant, and anti‐convulsion effects, possibly through GABAergic interaction pathways [24, 25, 26]. However, its antidepressant effects, along with its GABAergic interactions, are yet to be discovered.

Knowing the overall facts, the current study aimed to evaluate the antidepressant effect of PHY using two widely used mouse models. To determine the effects of PHY on male and female animals, we included both sexes of mice in this study. To understand the possible molecular mechanism behind this neurological effect of PHY, we combined it with a GABAergic agonist and/or an antagonist drug and a serotonin reuptake blocker and also performed *in silico* studies with GABA_A_ receptor‐responsive subunits and the serotonin transporter (5HT_1A_). Additionally, we also performed an in vitro GABA inhibition test using a GABA supplement of the test sample and/or reference drugs.

## 2 Martials and Methods

### 2.1 Chemicals and Reagents

Phytol (PHY, CAS: 7541‐49‐3; > 95% (HPLC: high‐performance liquid chromatography)) was bought from Sigma‐Aldrich, Germany, while the standard drug diazepam (DZP) and fluoxetine were kindly provided by Square Pharmaceuticals Ltd., Bangladesh, and flumazenil (FLU) was purchased from Centurion Healthcare Private Ltd., India, respectively. A GABA supplement, pregabalin (Gabarol150) was purchased from the local market (source: Advanced Chemical Industries Limited, Bangladesh). Reagent‐grade sodium acetate trihydrate, acetic acid, NaOH, and HCl were purchased from Merck (India). Tween 80 and sodium chloride (NaCl) required for this study were purchased from Merck (India).

### 2.2 Experimental Animals

Adult male and female *Mus musculus* (*Swiss* mice; avg. b.w. 24–30 g) purchased from the Animal House of Jahangirnagar University, Bangladesh, were randomly divided into different groups of 6 animals each. Before that, the animals were housed in standard conditions (temperature: 26°C ± 2°C, relative humidity: 65%) for 7 days. They had free access to standard food and water *ad libitum*. This study was approved by the Animal Ethics Committee of Khulna University (KUAEC‐2024‐03‐01).

### 2.3 Selection and Administration of Doses/Concentrations

The test doses for PHY (25, 50, and 75 mg/kg) in mice were selected according to previous studies [27, 28]. The control (vehicle: distilled water containing 0.9% NaCl and 0.5% tween 80) and PHY (all doses) were administered via oral gavage (p.o.), while DZP (2 mg/kg), FLUX (20 mg/kg) and FLU (0.1 mg/kg) were administered through intraperitoneal injection (i.p.). Before treatment, all animals were fasted overnight. The mice were randomly categorized into different groups, each comprising six animals (*n* = 6). Treatments were given 30 min before starting the study, followed by the below‐mentioned studies in the in vivo section. For the in vitro GABAergic activity study, the highest dose of PHY was converted to the high test concentration of 75 μg/mL, which was then serially diluted to 37.5, 18.75, 9.375, and 4.6875 μg/mL. Similarly, DZP was diluted from 2 to 0.125 μg/mL, while FLU was diluted from 0.1 to 0.00625 μg/mL, respectively, and FLUX was prepared at 20 μg/mL. The combination groups were prepared with PHY‐75 and all tested concentrations.

### 2.4 In Vitro GABA Activity Analysis

The inhibitory neurotransmitter γ‐aminobutyric acid (GABA) was first described in the early 1950s as a major amine in the brain, which was discovered as a potential therapeutic target for depression. Defects in GABA function and its receptors are evident to contribute to depressive disorders. For example, major depressive disorder (MDD) is associated with lower concentrations of GABA. Antidepressant drugs can normalize it. It is evident that agents/drugs that can boost or suppress GABA_A_ receptors may have antidepressant effects. It has been seen that an inhibition of GABA levels in the brain interneurons can produce antidepressant effects [29]. Thus, it is necessary to check the current test drug to see if it has GABA‐modulatory effects or not. Knowing the overall facts, this study was conducted according to an earlier described model by Jinnarak and Teerasong [30] with some modifications. For this, a stock solution of 2 g/L of GABA was prepared by dissolving 200 mg of GABA (GABA dietary supplement (Pregabalin)) in 100 mL of distilled water. The solution was then filtered and kept in a conical flask. A volume of 1 mL of acetate buffer was added to 1 mL of GABA solution and kept in individual test tubes containing 1 mL of test sample (PHY) with different concentrations (4.6875–75 μg/mL). As positive controls, DZP (GABA agonist) and FLU (GABA antagonist) were used at a specified series of concentrations as mentioned above. The final volume of 5 mL was adjusted with double‐distilled water (DDW). DDW was considered a negative control for this assay. To prepare 0.5 M acetate buffer at pH of 3.8, 50 mL of 0.5 M (3.402 g) sodium acetate trihydrate was mixed with 450 mL of 0.5 M acetic acid (30.026 g). The pH of the solution was adjusted to 3.8 by the addition of NaOH or HCl. Then the reaction mixture was vortexed for 20 s and observed for color change. Finally, the optical density of the mixture was measured at 620 nm using a colorimeter (LT‐114, India). The percentage GABA inhibitory activity was determined by using the following formula:

$$\begin{array}{c} \%\text{GABA inhibition}=\\ 100—\left[\left\{\left(\mathrm{OD}\;\text{of Control}—\mathrm{OD}\;\text{of Test}\right)÷\mathrm{OD}\;\text{of Control}\right\}\times 100\right] \end{array}$$

The half‐minimal inhibitory concentration (IC_50_) values for the test sample and/or standards were also determined using nonlinear regression analysis in GraphPad Prism.

### 2.5 In Vivo Approach

#### 2.5.1 Antidepressant Effect Study in Swiss Mice

##### 2.5.1.1 Study Design

In this study, a total of 60 male and 60 female *Swiss* mice were randomly divided into 20 groups. We used both sexes to determine sex‐dependent effects of PHY and its combinations in animals. For this, the first 10 groups were comprised of male animals (*n* = 6), while the other 10 groups contained female animals (*n* = 6). Treatment groups and their details have been shown in Table 1.

**TABLE 1:** Treatment groups with their details at a 10 mL/kg volume of administration.

Treatment groups	Description	Administration design
***Individual groups***		
Control	Vehicle: Distilled water containing 0.9% NaCl and 0.5% tween 80	p.o., At a time
DZP‐2	Diazepam (GABA agonist reference drug) at 2 mg/kg	i.p., At a time
FLU‐0.1	Flumazenil (GABA antagonist reference drug) at 0.1 mg/kg	i.p., At a time
FLUX‐20	Fluoxetine (Standard) at 20 mg/kg	p.o. At a time
PHY‐25	Phytol (test sample) at 25 mg/kg	p.o., At a time
PHY‐25	Phytol (test sample) at 50 mg/kg	p.o., At a time
PHY‐25	Phytol (test sample) at 75 mg/kg	p.o., At a time
***Combination groups***		
DZP‐2 + FLU‐0.1	Diazepam 2 mg/kg + Flumazenil 0.1 mg/kg	i.p. + i.p., One followed by another
PHY‐75 + DZP‐2	Phytol 75 mg/kg + Diazepam 2 mg/kg	p.o. + i.p., One followed by another
PHY‐75 + FLU‐0.1	Phytol 75 mg/kg + Flumazenil 0.1 mg/kg	p.o. + i.p., One followed by another
PHY‐75 + FLUX −20	Phytol 75 mg/kg + Fluoxetine 20 mg/kg	p.o. + p.o., One followed by another
PHY‐75 + DZP‐2 + FLU‐0.1	Phytol 75 mg/kg + Diazepam 2 mg/kg + Flumazenil 0.1 mg/kg	p.o. + i.p. + i.p., One followed by another

*Note:* Control: Vehicle (distilled water containing 0.9% NaCl and 0.5% tween 80); (*n* = 6).

Abbreviations: DZP, Diazepam; FLU, Flumazenil; FLUX, Fluoxetine; i.p., Intraperitoneal; PHY, Phytol.

Thirty minutes after all treatments, each animal was subjected to the following test protocols. Between each test, an animal was given a 2‐min break to face the new apparatus.

##### 2.5.1.2 Immobility Time Assessment Through the Tail‐Suspension Test

This study was performed using the method described by Steru et al. [31], with slight modifications. Briefly, 30 min after treatment, each mouse was hung using surgical tape attached to its tail and a hard plane just above 22 cm from the floor. Each animal was hung 5 cm from the end of its tail on a surface and observed for 5 min. Immobility time was recorded by using a stopwatch.

##### 2.5.1.3 Immobility Time Assessment Through Forced‐Swimming Test

This study was done according to the method described by Porsolt et al. [32], with slight modifications. Briefly, the apparatus consisted of a large glass beaker (3‐l capacity). The beaker was filled with water (25°C ± 1°C) to a depth of 15 cm. Animals that passed a pretest swimming session of 5 min each before the final test were considered for this study. This test is followed by a previous study for 5 min. Within this time, the duration of immobility was recorded by using a stopwatch. Animals did not show escape except for movements necessary to keep their heads out of the water and were considered immobile [33].

### 2.6 Statistical Analysis

Values are expressed as the mean ± SD (standard deviation). One‐way analysis of variance (ANOVA) followed by a Tukey multiple comparison test at 95% confidence intervals using GraphPad Prism software (version: 9.5, San Diego, USA) considered *p <* 0.05. We performed Z‐score normalization (standardizing based on the mean and standard deviation) of the obtained data. We analyzed and compared the immobility time in seconds in both tests of the test drug (PHY) in comparison to its different doses and/or controls (e.g., vehicle, DZP, FLU and FLUX).

### 2.7 *In Silico* Approach

#### 2.7.1 Preparation of Ligands

**FIGURE 1:**
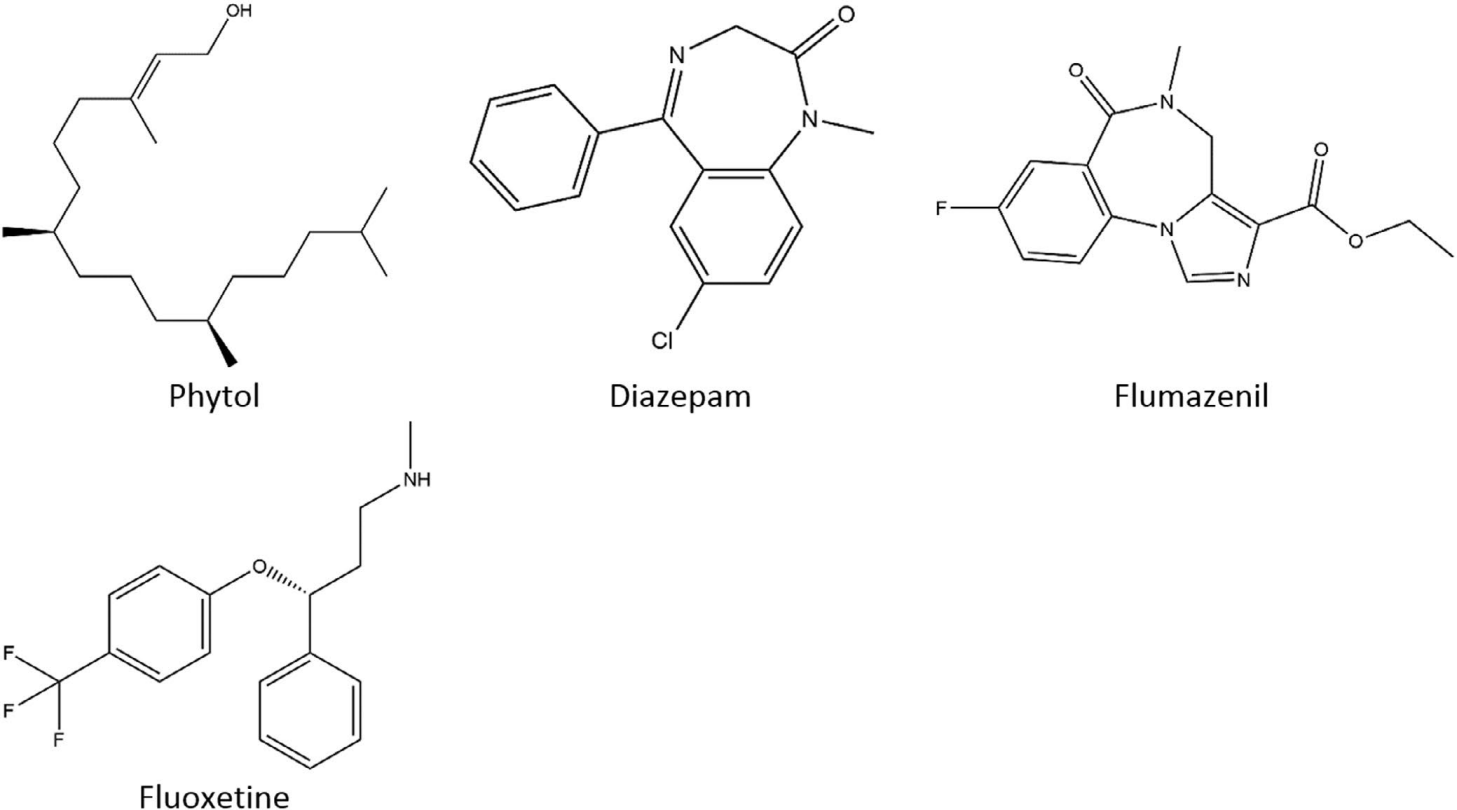
The 2D chemical structures of the test sample and the reference drugs.

The three‐dimensional (3D) conformers of compound PHY (PubChem ID: 5280435), standard drug DZP (PubChem ID: 3016), FLU (PubChem ID: 3373), and FLUX (PubChem ID: 3386) were retrieved in SDF format from the PubChem chemical database (https://pubchem.ncbi.nlm.nih.gov/, accessed on August 6, 2024). Subsequently, the 3D conformers of these compounds underwent energy minimization and were saved as SDF files using the Chem3D 16.0 software suite to facilitate molecular docking studies. The two‐dimensional representations of these chemical agents are illustrated in Figure 1.

#### 2.7.2 Protein Selection and Preparation

We have selected the α1, α2, α3, α5, and γ2 subunits of the human GABA_A_ receptor as targets for molecular docking and visualization studies based on the literature [15, 16, 34, 35, 36]. We also selected the 5HT_1A_ receptor, which is liable for the depression pathway [37]. 3D structures of the 5HT_1A_ (PDB: 3GWW, Chain A) and GABA_A_ α3 (PDB: 8G4X (Chain G [auth C]) α1, γ2 (PDB: 6X3X, Chain D and E), and α5 (PDB: 8BHK, Chain A)) were collected from the Protein Data Bank (RCSB PDB) (https://www.rcsb.org/) and α2 (AlphaFold ID: AF‐P26048‐F1, Uniprot: P26048) was gathered from the Protein Structure Database (AlphaFold) (https://alphafold.com/). The receptors were optimized to prevent docking interference by removing unwanted protein chains, lipids, water molecules, and heteroatoms [38]. This optimization process was carried out using Discovery Studio Visualizer v24.1.0.23298. Next, the protein structure underwent energy minimization using the Swiss‐PDB Viewer software (version 4.1.0) [39].

#### 2.7.3 Docking Protocol and Non‐bond Interactions

We performed molecular docking of phytol and other selected ligands with the GABA_A_ receptor (α1, α2, α3, and α5 subunits) and 5HT_1A_ using the PyRx software suite to predict the active binding potential of the compounds at the receptor sites. The docking process involved enclosing both receptors and the ligand within a grid box, with dimensions set to center at −0.33 × −0.65 × −6.75 Å for GABA_A_ α2, 138.04 × 174.99 × 132.78 Å for GABA_A_ α3, 20.01 × 18.54 × 17.29 Å for GABA_A_ α1, γ2, and maximize for GABA_A_ α5 and 23.69 × 32.15 × 35.37 Å for 5HT_1A_ along the x‐, y‐, and z‐axes, respectively, and running the calculation over 200 steps. Docking results were saved in CSV format, and the optimal pose (top‐ranked docking conformation based on ligand binding affinity and RMSD lower and upper bounds) was extracted in PDB format for subsequent binding interaction analysis. These interactions, encompassing amino acid residues, bond types, hydrogen bond lengths, and other bond characteristics for each ligand‐receptor interaction, were meticulously examined using the Discovery Studio Visualizer (v21.1.0.20298) [40].

#### 2.7.4 Pharmacokinetics, Drug‐Likeness, and Toxicity Prediction Properties

Drug‐likeness represents a qualitative assessment employed in drug research and development to evaluate the behavior of chemical entities concerning various drug‐like parameters, including bioavailability, and is closely associated with ADMET. The physicochemical properties and pharmacokinetic characteristics of PHY were predicted using the SwissADME [41], pkCSM [42], and ADMETlab 3.0 [43] online tools. Additionally, the toxicity profile of PHY was assessed using the Protox 3.0 online server, which was primarily utilized to investigate parameters such as hepatotoxicity, carcinogenicity, immunotoxicity, mutagenicity, and cytotoxicity. Data sourced from PubChem, including SMILES (Simplified Molecular Input Line‐Entry System) notations, were input into SwissADME, pkCSM, ADMETlab 3.0, and ProTox 3.0 online tools to analyze the specified properties.

## 3 Results

### 3.1 In Vitro Findings

The control group (DDW) exhibited a negligible GABA inhibitory effect (4.75% ± 0.01%), in comparison to PHY and DZP, which concentration‐dependently and significantly (*p* < 0.05) inhibited GABA in test tubes. At high concentrations, PHY (75 μg/mL) and DZP (2 μg/mL) exhibited GABA inhibition of 62.50% ± 0.03% and 72.50% ± 0.01%, respectively. In contrast, FLU concentration‐dependently but significantly (*p* < 0.05) reduced GABA inhibition in the test tubes, whereas at the low convention (0.00625 μg/mL) it showed GABA inhibition of 55.00% ± 0.02%. PHY 75 μg/mL combined with DZP also concentration‐dependently and significantly (*p* < 0.05) inhibited GABA, where at high concentration (PHY‐75 + DZP‐2) showed the highest GABA inhibition (92.50% ± 0.02%). In contrast, the PHY‐75 + FLU combination concentration‐dependently but significantly (*p* < 0.05) reduced the GABA inhibition in the test tubes, where the low concentration combination (PHY‐75 + FLU‐0.00625) exhibited the highest GABA inhibition of 72.50% ± 0.01%. The IC_50_ values calculated for PHY, DZP, FLU, PHY‐75 + DZP, and PHY‐75‐FLU are 46.75 ± 1.22, 0.87 ± 0.43, 0.005 ± 0.001, 0.03 ± 0.01, and 0.002 ± 0.001 μg/mL, respectively (Table 2).

**TABLE 2:** GABAergic activity of phytol and/or controls.

Treatment with concentration		Percentage GABAergic activity	IC_50_ [CI; R^2^]
Control (DDW)	1 mL	4.75 ± 0.01	—
PHY	4.6875 μg/mL	10.00 ± 0.01*	46.75 ± 1.22 μg/mL [42.40–52.63 μg/mL; 0.98]
	9.375 μg/mL	17.50 ± 0.01*	
	18.75 μg/mL	32.50 ± 0.01*	
	37.5 μg/mL	47.50 ± 0.02*	
	75 μg/mL	62.50 ± 0.03*	
DZP	0.125 μg/mL	17.50 ± 0.01*	0.87 ± 0.43 μg/mL [0.84–1.59 μg/mL; 0.95]
	0.25 μg/mL	27.50 ± 0.01*	
	0.5 μg/mL	37.50 ± 0.01*	
	1 μg/mL	57.50 ± 0.01*	
	2 μg/mL	72.50 ± 0.01*	
FLU	0.00625 μg/mL	55.00 ± 0.02*	0.005 ± 0.001 μg/mL [0.003–0.006 μg/mL; 0.94]
	0.0125 μg/mL	37.50 ± 0.02*	
	0.025 μg/mL	32.50 ± 0.02*	
	0.05 μg/mL	20.00 ± 0.01*	
	0.1 μg/mL	12.50 ± 0.01*	
PHY‐75 + DZP	0.125 μg/mL	70.00 ± 0.02*	0.03 ± 0.01 μg/mL [0.008–0.12 μg/mL; 0.93]
	0.25 μg/mL	77.50 ± 0.01*	
	0.5 μg/mL	80.00 ± 0.01*	
	1 μg/mL	87.50 ± 0.01*	
	2 μg/mL	92.50 ± 0.02*	
PHY‐75 + FLU	0.00625 μg/mL	72.50 ± 0.01*	0.002 ± 0.001 μg/mL [0.002–0.004 μg/mL; 0.95]
	0.0125 μg/mL	55.50 ± 0.02*	
	0.025 μg/mL	45.00 ± 0.01*	
	0.05 μg/mL	30.00 ± 0.02*	
	0.1 μg/mL	22.50 ± 0.02*	

*Note:* Values are the mean percentage ± SD (*n* = 3); One‐way ANOVA followed by Tukey multiple comparison test at 95% confidence intervals.

Abbreviations: CI, confidence of intervals; DZP, diazepam; FLU, flumazenil; IC_50_, 50% inhibitory concentration; *R* ^2^, co‐efficient of determination.

* *p <* 0.05 compared to the Control (Vehicle: Double distilled water) Phytol.

### 3.2 In Vivo Findings

#### 3.2.1 Immobility Time in Tail‐Suspension Test

For male animals, the control group exhibited IMT of 22.00 ± 1.10 s, while the standard agonist drug DZP (2 mg/kg, p.o.) had 94.33 ± 3.14 s and the antagonist drug FLU (0.1 mg/kg, i.p.) had 6.17 ± 1.17 s in TST. On the other hand, the SSRI drug FLUX (20 mg/kg, p.o.) demonstrated significantly (*p* < 0.05) reduced IMT (7.00 ± 2.89 s). PHY dose‐dependently and significantly (*p* < 0.05) reduced IMT values in animals, where at 75 mg/kg it showed an IMT value of 10.17 ± 2.13 s. PHY‐75 combined with DZP‐2 remarkably and significantly (*p* < 0.05) augmented the IMT values, while combined with FLU‐0.1 it reversed the situation. The PHY‐75 + DZP‐2 + FLU‐0.1 group exhibited an IMT value of 19.17 ± 3.92 s between the PHY‐75 + DZP‐2 and PHY‐75 + FLU‐0.1 groups. The combination treatment of PHY‐75 + FLUX resulted in slightly higher IMTs (10.00 ± 3.25 s). For female animals, the control group showed an augmented IMT value (24.00 ± 2.00 s), while DZP‐2 reduced this parameter (89.50 ± 7.50 s) compared to the male control group. In contrast, the antagonist group (FLU‐0.1) augmented IMT (7.50 ± 1.87 s) values compared to the male group. FLUX showed significantly (*p* < 0.05) decreased IMT values (7.17 ± 3.66 s). PHY dose‐dependently and significantly (*p* < 0.05) reduced IMT values in animals, where at 75 mg/kg it showed an IMT value of 10.83 ± 2.56 s. PHY‐75 combined with DZP‐2 also remarkably and significantly (*p* < 0.05) augmented the IMT values, while combined with FLU‐0.1 it reversed the situation. The PHY‐75 + DZP‐2 + FLU‐0.1 group exhibited an IMT value of 20.17 ± 3.54 s between the PHY‐75 + DZP‐2 and PHY‐75 + FLU‐0.1 groups. The PHY‐75 + FLUX group recorded a comparable IMT of 7.83 ± 2.14 s. Except for the DZP‐2 group, in all cases, female animals showed a slight augmented IMT value when compared to the male group animals (Table 3).

**TABLE 3:** Immobility time (sec) observed in tail‐suspension and forced‐swimming studies (sec) observed in different treatment groups of male and female animals.

Treatment groups	IMT‐TST (Sec)		IMT‐FST (Sec)	
	Male	Female	Male	Female
***Individual groups***				
Control	22.00 ± 1.10	24.00 ± 2.00	31.33 ± 5.65	31.17 ± 5.46
DZP‐2	94.33 ± 3.14	89.50 ± 7.50	83.33 ± 5.17	77.17 ± 3.19
FLU‐0.1	6.17 ± 1.17*^abc^	7.50 ± 1.87*^ac^	5.67 ± 1.63*^abc^	6.50 ± 1.87*^abc^
FLUX	7.00 ± 2.89*	7.17 ± 3.66*	7.33 ± 2.07*	8.33 ± 3.61*
PHY‐25	22.67 ± 3.67^a^	24.50 ± 4.23^a^	32.17 ± 2.86^a^	35.17 ± 5.12^a^
PHY‐50	16.33 ± 3.44*^a^	19.67 ± 4.46*^a^	15.17 ± 4.40*^a^	18.50 ± 4.18*^a^
PHY‐75	10.17 ± 2.13*^a^	10.83 ± 2.56*^a^	12.17 ± 3.97*^a^	14.50 ± 4.85*^a^
***Co‐treatment groups***				
DZP‐2 + FLU‐0.1	40.83 ± 7.05*^a^	42.33 ± 5.35*^a^	35.17 ± 3.71*^a^	37.83 ± 3.60^a^
PHY‐75 + DZP‐2	89.00 ± 8.27^a^	91.67 ± 6.56	75.67 ± 10.73^a^	78.50 ± 10.21
PHY‐75 + FLU‐0.1	10.83 ± 2.71*^a^	12.50 ± 3.45*^a^	9.33 ± 4.18*^ac^	11.17 ± 4.02*^ac^
PHY‐75 + DZP‐2 + FLU‐0.1	19.17 ± 3.92*^a^	20.17 ± 3.54*^a^	26.67 ± 3.44*^a^	27.67 ± 3.33*^a^
PHY‐75 + FLUX	10.00 ± 3.25*	7.83 ± 2.14*	7.17 ± 2.71*^b^	8.00 ± 2.89*^b^

*Note:* Values are the mean ± SD (*n* = 6); One‐way ANOVA followed by Tukey multiple comparison test; *p <* 0.05 compared to the *Control (vehicle), ^a^DZP‐2 (diazepam 2 mg/kg), ^b^FLUX (Fluoxetine 20 mg/kg) and ^c^PHY‐75 (phytol 75 mg/kg) group.

Abbreviations: FLU‐0.1, Flumazenil 0.1 mg/kg; FST, Forced‐swimming test; IMT, Immobility time (Sec); TST, Tail‐suspension test.

#### 3.2.2 Immobility Time in Forced‐Swimming Test

For male animals, in the FST, the control group exhibited IMT of 31.33 ± 5.65 s. The standard agonist group DZP‐2 significantly (*p* < 0.05) increased IMT (83.33 ± 5.17 s), while the antagonist drug FLU (0.1 mg/kg) significantly (*p* < 0.05) reduced (5.67 ± 1.63 s) this parameter when compared to the control group. In addition, FLUX showed a significantly (*p* < 0.05) reduced IMT of 7.33 ± 2.07 s. As shown in the TST, in this case, PHY also dose‐dependently reduced this parameter significantly (*p* < 0.05), where at high dose it showed an IMT value of 12.17 ± 3.97 s. The highest number IMT value was observed in the DZP‐2 group, while the lowest was observed in the FLU‐0.1 group (5.67 ± 1.63 s). In this case, the combined group PHY‐75 + DZP‐2 + FLU‐0.1 also exhibited an IMT value between the PHY‐75 + DZP‐2 and PHY‐75 + FLU‐0.1 groups. The combination treatment of PHY‐75 + FLUX resulted in a closely comparable IMT of 7.17 ± 2.71 s. For female animals, the control group showed close IMT values (31.17 ± 5.46 s), while DZP‐2 reduced this parameter (77.17 ± 3.19 s) when compared to the male control group. FLUX treatment similarly demonstrated a significantly (*p* < 0.05) decreased IMT of 8.33 ± 3.61 s. In this case, the FLU‐0.1 group also augmented IMT (6.50 ± 1.87 s) values compared to the male group. PHY dose‐dependently and significantly (*p* < 0.05) reduced IMT values in animals, where at 75 mg/kg it showed an IMT value of 27.67 ± 3.33 s. The combined group PHY‐75 + DZP‐2 also remarkably and significantly (*p* < 0.05) augmented the IMT values, while with PHY‐75 + FLU‐0.1 it reversed the situation. The PHY‐75 + DZP‐2 + FLU‐0.1 group exhibited an IMT value of 27.67 ± 3.33 s between the PHY‐75 + DZP‐2 and PHY‐75 + FLU‐0.1 groups. However, the PHY‐75 + FLUX group recorded a comparable IMT of 8.00 ± 2.89 s. Except for the control and DZP‐2 groups, in all cases, female animals showed a slight augmentation in IMT value when compared to the male group animals (Table 3).

### 3.3 *In Silico* Findings

#### 3.3.1 PHY, DZP And FLU With GABA_A_ (α2) Receptor Interactions

The molecular docking analysis revealed that DZP and PHY both showed a binding affinity of −6.5 kcal/mol with the GABA_A_ (α2) receptor. DZP formed a hydrogen bond with TYR A: 187 (2.92 Å) and established pi‐cation, pi‐pi t‐shaped, and pi‐alkyl interactions with TYR A: 237, PHE A: 127, HIS A: 129, and TYR A: 187, respectively. PHY similarly formed a hydrogen bond with SER A: 232 (2.77 Å) and hydrophobic interactions with ILE A: 230, PHE A: 127, HIS A: 129, and TYR A: 237. In comparison, FLU exhibited a slightly lower binding affinity of −5.5 kcal/mol, forming a hydrogen bond with PHE A: 323 (2.48 Å) along with unique fluorine interactions with PHE A: 323 and ALA A: 322, as well as alkyl interactions with VAL A: 435, VAL A: 319, and LEU A: 428.

#### 3.3.2 PHY, DZP And FLU With GABA_A_ (α3) Receptor Interactions

In this study, DZP showed a strong binding affinity of −6.8 kcal/mol, forming three hydrogen bonds with ASN C: 112 (2.22 Å), THR C: 138 (2.32 Å), and LYS C: 141 (2.67 Å), along with amide‐pi stacked and pi‐alkyl interactions with ASN C: 140, ARG C: 156, and LEU C: 142. PHY also showed the highest affinity at −7.2 kcal/mol, creating one hydrogen bond with PRO C: 139 (1.78 Å) and engaging in pi‐sigma, alkyl, and pi‐alkyl interactions with PHE C: 89, ARG C: 156, and LEU C: 142. FLU, with a binding affinity of −6.9 kcal/mol, formed three hydrogen bonds with THR C: 137 (2.41 Å), THR C: 138 (2.31 Å), and PRO C: 139 (2.53 Å), in addition to amide‐pi stacked and pi‐alkyl interactions with ASN C: 140 and ARG C: 156.

#### 3.3.3 PHY, DZP And FLU With GABA_A_ (α1 and γ2 Subunits) Receptor Interactions

In this molecular docking study, DZP exhibited a binding affinity of −6.6 kcal/mol, forming a hydrogen bond with MET E: 331 (2.82 Å) and additional interactions, including an attractive charge interaction with GLU E: 313, a hydrogen bond with MET E: 331, and an alkyl interaction with VAL E: 312. PHY showed a binding affinity of −4.7 kcal/mol, primarily engaging in pi‐sigma interactions with PHE E: 339 and PHE E: 306, and multiple alkyl interactions with ALA E: 335, ALA E: 342, MET E: 331, and ILE E: 305, as well as a pi‐alkyl interaction with PHE E: 338. FLU displayed the highest affinity at −6.8 kcal/mol, forming two hydrogen bonds with PRO E: 243 (2.71 Å) and ASP D: 287 (3.01 Å), and additional pi‐pi stacked and pi‐alkyl interactions with TYR D: 294, ALA D: 291, TRP D: 288, and PRO E: 243.

#### 3.3.4 PHY, DZP And FLU With GABA_A_ (α5) Receptor Interactions

In this docking analysis, DZP showed a binding affinity of −5.3 kcal/mol, forming three hydrogen bonds with ASN A: 155 (2.16 Å), GLU A: 153 (2.11 Å), and PRO A: 143 (2.84 Å), as well as pi‐anion and pi‐donor hydrogen bond interactions with ASP A: 145 and GLU A: 153, respectively. On the other hand, PHY displayed a lower binding affinity of −3.9 kcal/mol, establishing two hydrogen bonds with ASN A: 155 (2.40 Å) and PRO A: 143 (2.91 Å). In contrast, FLU exhibited the strongest binding affinity at −6.2 kcal/mol, forming three hydrogen bonds with SER A: 110 (2.53 Å), ARG A: 120 (2.33 Å), and THR A: 99 (2.62 Å), along with hydrophobic interactions, including a fluorine interaction with PRO A: 100, an alkyl interaction with ALA A: 112, and a pi‐alkyl interaction with LEU A: 134.

#### 3.3.5 PHY, And FLUX With 5HT_1A_ Receptor Interactions

In the molecular docking analysis with the 5HT_1A_ receptor, FLUX exhibited the highest binding affinity (−7.9 kcal/mol) among the selected ligands. FLUX interacted with the receptor through multiple interactions, including a halogen bond with ILE A: 410, pi‐sigma interaction with PHE A: 494, and pi‐pi stacked interactions with TRP A: 406 AA residues. Additionally, FLUX engaged in alkyl interactions with ILE A: 410 and pi‐alkyl interactions with ARG A: 487, ILE A: 491, and PHE A: 414 AA residues. On the other hand, PHY showed a slightly lower binding affinity of −6.7 kcal/mol. PHY did not form any HBs but exhibited a range of pi‐sigma and alkyl interactions. PHY interacted with TRP A: 406 and PHE A: 414 via pi‐sigma interactions and established alkyl bonds with ILE A: 491 and ARG A: 487. PHY also displayed several pi‐alkyl interactions, specifically with PHE A: 96, TRP A: 99, TRP A: 406, PHE A: 414, and PHE A: 494.

**FIGURE 2:**
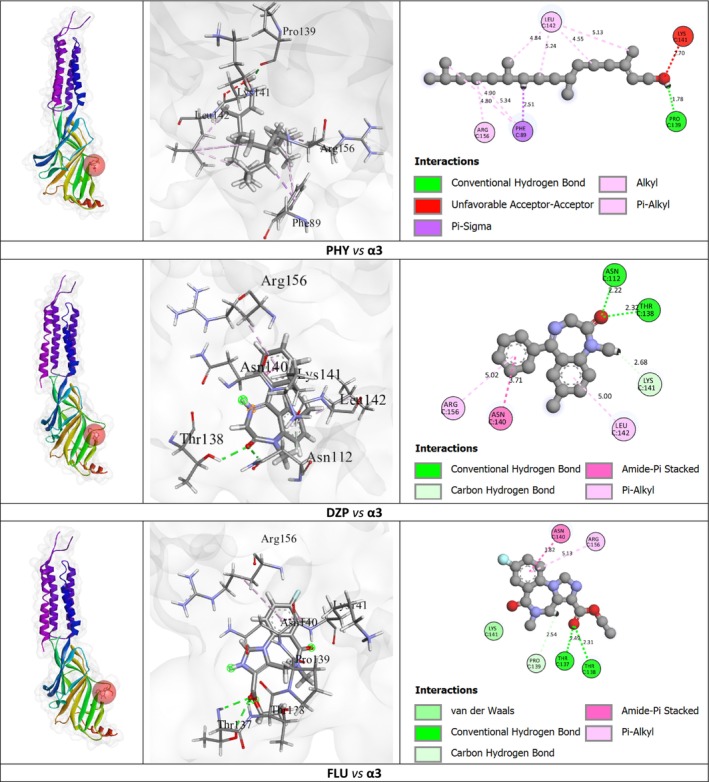
The 2D and 3D visualizations of the test sample and the reference drug interaction with the GABA_A_ receptor (α3 subunit).

Details of the binding affinity, hydrogen bonds, amino acid residues, bond lengths, and other interaction bonds among amino acid residues of PHY, FLU, and DZP in their interactions with the GABA_A_ receptor are presented in Table 4. Figure 2 illustrates the 2D and 3D structures of the binding pockets, along with the interacting residues and bond types of PHY, FLU, and DZP with the GABA_A_ receptor (α3 subunit).

**TABLE 4:** Molecular docking scores of phytol, diazepam, flumazenil, and fluoxetine against the GABA_A_ and 5HT_1A_ receptor.

Ligands	Receptors (PDB)	BA (kcal/mol)	No of HB	Amino Acid (AA) Residues	
				Hydrogen bond with length (Å)	Hydrophobic bonds (Type)
DZP	GABA_A_ (α2)	−6.5	1	TYR A: 187 (2.92)	TYR A: 237 (pi‐cation), PHE A: 127 (pi‐pi t‐shaped), ILE A: 230 (alkyl), HIS A: 129 (pi‐alkyl), TYR A: 187 (pi‐alkyl)
PHY		−6.5	1	SER A: 232 (2.77)	ILE A: 230 (alkyl), PHE A: 127 (pi‐alkyl), HIS A: 129 (pi‐alkyl), TYR A: 187 (pi‐alkyl), TYR A: 237 (pi‐alkyl)
FLU		−5.5	1	PHE A: 323 (2.48)	PHE A: 323 (fluorine), ALA A: A322 (fluorine), VAL A: 435 (alkyl), VAL A: 319 (alkyl), LEU A: 428 (pi‐alkyl)
DZP	GABA_A_ (α3)	−6.8	3	ASN C: 112 (2.22), THR C: 138 (2.32), LYS C: 141 (2.67)	ASN C: 140 (amide‐pi stacked), ARG C: 156 (pi‐alkyl), LEU C: 142 (pi‐alkyl)
PHY		−7.2	1	PRO C: 139 (1.78)	PHE C: 89 (pi‐sigma), ARG C: 156 (alkyl), LEU C: 142 (alkyl), PHE C: 89 (pi‐alkyl)
FLU		−6.9	3	THR C: 137 (2.41), THR C: 138 (2.31), PRO C: 139 (2.53)	ASN C: 140 (amide‐pi stacked), ARG C: 156 (pi‐alkyl)
DZP	GABA_A_ (α1 and γ2 subunits)	−6.6	1	MET E: 331 (2.82)	GLU E: 313 (attractive charge), MET E: 331 (carbon hydrogen bond), VAL E: 312 (alkyl)
PHY		−4.7	—	—	PHE E: 339 (pi‐sigma), PHE E: 306 (pi‐sigma), ALA E: 335 (alkyl), ALA E: 342 (alkyl), MET E: 331 (alkyl), ILE E: 305 (alkyl), PHE E: 338 (pi‐alkyl)
FLU		−6.8	2	PRO E: 243 (2.71), ASP D: 287 (3.01)	TYR D: 294 (pi‐pi stacked), ALA D: 291 (alkyl), TRP D: 288 (pi‐alkyl), PRO E: 243 (pi‐alkyl)
DZP	GABA_A_ (α5) (8BHK)	−5.3	3	ASN A: 155 (2.16), GLU A: 153 (2.11), PRO A: 143 (2.84)	ASP A: 145 (pi‐anion), GLU A: 153 (pi‐donor hydrogen bond)
PHY		−3.9	2	ASN A: 155 (2.40), PRO A: 143 (2.91)	—
FLU		−6.2	3	SER A: 110 (2.53), ARG A: 120 (2.33), THR A: 99 (2.62)	PRO A: 100 (fluorine), ALA A: 112 (alkyl), LEU A: 134 (pi‐alkyl)
FLUX	5HT_1A_ (3GWW)	−7.9	—	—	ILE A: 410 (halogen), PHE A: 494 (pi‐Sigma), TRP A: 406 (pi‐pi Stacked), TRP A: 406 (pi‐pi stacked), ILE A: 410 (alkyl), ARG A: 487 (pi‐alkyl), ILE A: 491 (pi‐alkyl), PHE A: 414 (pi‐alkyl)
PHY		−6.7	—	—	TRP A: 406 (pi‐Sigma), PHE A: 414 (pi‐sigma), ILE A: 491 (alkyl), ARG A: 487 (alkyl), PHE A: 96 (pi‐alkyl), TRP A: 99 (pi‐alkyl), TRP A: 406 (pi‐alkyl), PHE A: 414 (pi‐alkyl), PHE A: 494 (pi‐alkyl)

Abbreviations: BA, Binding affinity; DZP, Diazepam; FLU, Flumazenil; FLUX, Fluoxetine; GABA_A_, Gamma‐aminobutyric acid type A receptor; HB, Hydrogen bonds; PHY, Phytol.

### 3.4 Pharmacokinetic Profile

The *in silico* evaluation of ADMET (absorption, distribution, metabolism, excretion, and toxicity) properties remains a cornerstone in contemporary drug discovery, facilitating the prediction of pharmacokinetic profiles and optimizing bioavailability [44]. This evaluation encompasses a spectrum of parameters such as molecular weight (MW), hydrogen bond acceptors (HBA), hydrogen bond donors (HBD), polar surface area, consensus log P, molar refractivity (MR), and water solubility to rigorously assess the physicochemical characteristics of drug candidates [45, 46]. *In silico* predictions indicate that PHY demonstrates a superior physicochemical profile compared to FLU and DZP. All three ligands possess a molecular weight (MW) below 500 g/mol, with acceptable ranges for hydrogen bond acceptors (HBA), hydrogen bond donors (HBD), molar refractivity (MR), and topological polar surface area (TPSA), aligning well with drug‐likeness standards. PHY is moderately soluble, while DZP and FLU are both soluble in water. Pharmacokinetic predictions reveal that PHY has low gastrointestinal (GI) absorption but can effectively cross the blood–brain barrier (BBB), which may contribute to central nervous system effects. Key pharmacokinetic attributes such as P‐glycoprotein (P‐gp) substrate status, inhibition profiles of cytochrome P450 enzymes (CYP1A2, CYP2C19, and CYP3A4), and skin permeability (log K_p_) are within favorable ranges for all ligands, with a consistent bioavailability score of 0.55.

**FIGURE 3:**
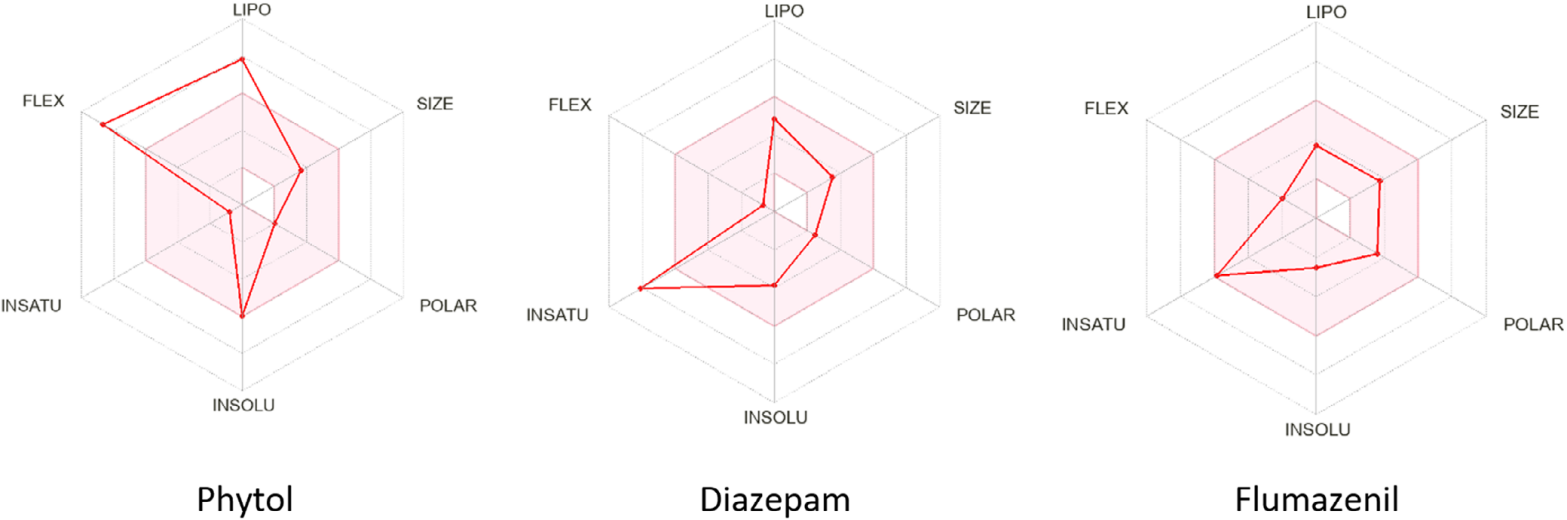
Summary of physicochemical or drug likeness properties of selected compounds [The colored zone is the suitable physicochemical space for oral bioavailability; SIZE: 150 g/mol<MV < 500 g/mol; INSOLU (Insolubility): −6 < log S (ESOL) < 0; LIPO (Lipophilicity): −7 < XLOGP3 < +5.0; INSATU (In saturation): 0.25 < Fraction Csp3 < 1; POLAR (Polarity): 20 Å^2^ < TPSA< 130 Å^2^; FLEX (Flexibility): 0 < num. Rotatable bonds < 9].

Toxicity assessments indicate high LD_50_ values (5000 mg/kg for PHY, 48 mg/kg for DZP, and 1300 mg/kg for FLU), suggesting that PHY is relatively safer at high doses. The compounds are assigned toxicity classes 5, 2, and 4, respectively. None of the ligands exhibit carcinogenicity, mutagenicity, hepatotoxicity, or immunotoxicity; however, DZP shows cytotoxicity, distinguishing it from PHY and FLU, which remain inactive in cytotoxicity predictions. Detailed values for various physicochemical, pharmacokinetic, and toxicity parameters of the ligands are presented in Table 5. A graphical presentation is included in Figure 3.

**TABLE 5:** The documented summary of the *in silico* physicochemical, pharmacokinetic, and toxicity properties of the test sample and the reference standards.

Properties	Parameters	Report/Predicted value		
		PHY	DZP	FLU
Physicochemical	MF	C_20_H_40_O	C_16_H_13_CIN_2_O	C_15_H_14_FN_3_O_3_
	MW (g/mol)	296.53	284.74	303.29
	H‐bond acceptors	1	2	5
	H‐bond donor	1	0	0
	TPSA (Å^2^)	20.23	32.67	64.43
	Consensus Log P	6.25	2.97	1.73
	MR	98.94	87.95	79.47
	Solubility	Moderately soluble	Soluble	Soluble
Pharmacokinetics	GI absorption	Low	High	High
	BBB permeability (log BB)	0.793	0.331	−0.205
	Pgp substrate	No	Yes	No
	CYP1A2 int	Yes	Yes	Yes
	CYP2C19 int	No	Yes	No
	CYP2D6 int	No	No	No
	CYP3A4 int	No	No	No
	log Kp (cm/s)	−2.29	−5.91	−7.44
Druglikeness	BIO Score	0.55	0.55	0.55
	Lipinski	Yes; 1 violation: MLOGP > 4.15	Yes; 0 violation	Yes; 0 violation
Toxicity	LD_50_ (mg/kg)	5000	48	1300
	Toxicity class	5	2	4
	Hepatotoxicity	Inactive	Inactive	Inactive
	Carcinogenicity	Inactive	Inactive	Inactive
	Immunotoxicity	Inactive	Inactive	Inactive
	Mutagenicity	Inactive	Inactive	Inactive
	Cytotoxicity	Inactive	Active	Inactive

Abbreviations: BBB, Blood–brain barrier; BIO Score, Bioavailability Score; CYP2C19 int., CYP2C19 inhibitor; DZP, Diazepam; FLU, Flumazenil; GI, Gastrointestinal; LD_50_, Lethal dose 50; MF, Molecular formula; MR, Molar refractivity; MW, Molecular weight; PHY, Phytol.

## 4 Discussion

The GABA receptor is the key target for the development of anxiolytic and/or depressive medications [47]. GABA is an important inhibitory neurotransmitter involved in brain growth and function, as well as preserving the balance between neuronal activation and inhibition in the CNS [48]. Primarily, the allosteric regulation of the BDZ site is the most commonly utilized hypnotic that influences GABA systems [18]. However, BDZs produce tolerance as well as physical dependency in patients. Nowadays, conventional antidepressants have minimal effectiveness and dietary limitations, and some people are nonresponsive entirely [49]. As a consequence, the development of novel, effective, and safer antidepressants is an urgent priority.

The GABA agonist drug DZP downregulates the GABA_A_ receptor, especially its α1 subunit, thereby increasing GABA concentration in the brain, which is revered by the GABA antagonist drug FLU [50]. Our in vitro study demonstrates that both PHY and DZP concentration‐dependently and significantly (*p* < 0.05) increased the GABA inhibitory capacity, while FLU and its combinations with PHY reversed the situation. On the other hand, in the preclinical study, FST and TST have been established to examine the effects of antidepressants on animals [51]. For widely recognized antidepressants, the TST shows consistency, but for medications with unclear mechanisms of action, its accuracy rate is questionable [52]. On the other hand, FST has a high degree of predictive validity, which reveals the possible acute antidepressant activities of novel compounds [53]. In this study, in both cases, we have also seen that FLU‐0.1 alone or with PHY‐75 reduced IMT values in experimental animals, suggesting its antidepressant effects. On the other hand, the GABA‐agonist drug DZP alone or in combination with PHY‐75 augmented IMT values significantly. PHY alone exhibited an action between the FLU and DZP in animals, where it dose‐dependently reduced the IMT values in animals. An earlier report by Costa et al. [27]; [28] also suggests that PHY dose‐dependently enhanced the number of square crosses, rearing, and grooming compared to the control group in an open‐field study. It also enhanced the performance time in the rota‐rod test, increased light‐spent time in the dark–light study, and increased the number of entries in the open arms as well as the time of permanence in the open in the elevated‐plus‐maze test. In this study, the authors checked the anxiolytic effect of PHY with or without the standard drugs DZP and FLU, where PHY exhibited dose‐dependent motor activity‐enhancing capacity in animals. Another study reports that PHY exhibited an antidepressant‐*like* effect, possibly by reducing oxidative stress in the brains of experimental animals [54]. Besides TST, FST also allows us to record IMT; a reduction of that generally occurs means reduced motor activity in animals [55]. FLU improves the psychomotor performance of animals, while DZP may reduce it [56]. Deficits in the storage or retrieval of motor memories contribute to sensorimotor deficits and implicate frontoparietal networks in animals [57]. Sathya et al. [23] demonstrated that PHY and PHY‐loaded poly lactic‐co‐glycolic acid nanoparticles (Phytol‐PLGANPs) have potent anti‐amnesic effects and multi‐faceted neuroprotective potential against scopolamine‐induced memory dysfunction in animals. In this study, the authors demonstrate that PHY may produce that effect through attenuating cholinesterase activity, oxidative stress, and apoptosis in animals. A reduction of cholinesterase activity is evident to improve motor activity in animals [58]. In our study, we have also seen that PHY dose‐dependently reduced IMT values in both tests, which was seen in the GABA antagonist drug FLU group. In contrast, the sedative drug DZP significantly augmented IMT values in animals. PHY co‐treated with these drugs also modulated IMT values significantly, suggesting agreement with the previous reports on PHY in experimental animals, and it is possible that PHY may exert its antidepressant effects through multiple pathways.

SSRIs are commonly prescribed antidepressants, primarily used to treat depression and anxiety disorders. They work by increasing serotonin levels in the brain and are preferred for their safety, efficacy, and relatively mild side effects [59, 60]. However, FST and TST are widely used animal models for evaluating antidepressant‐like activity, particularly in relation to the serotonin pathway, which plays a crucial role in mood regulation and is often targeted in depression therapies [61, 62]. In these FST and TST tests, FLUX and PHY significantly (*p* < 0.05) reduced IMT, indicating potential antidepressant effects. The combination treatments of PHY‐75 with FLUX did not significantly change IMT values, suggesting that PHY may synergize with or enhance the effects of FLUX in reducing IMT. According to the results, PHY has dose‐dependent efficacy, and FLUX has strong antidepressant effects.

Preclinical and clinical research has revealed that depression has been associated with GABAergic dysregulation [16]. Additionally, we observed in this study that the combination of DZP, FLU, and PHY had sex‐dependent impacts on mice. It might be related to the effect of gonadal hormonal changes in male and female animals [63]. Both estrogen receptors α and β have been linked to GABAergic cell modulation and amygdala GABA_A_ cell densities that may be a source of anxiety‐like behavior in female animals [64]. Estrogen quickly changes dopaminergic neurotransmission by membrane‐bound receptor GABAergic neurons in animals, implicating anxiety conditions (Favilla et al. 2008). The abnormal regulation of inhibitory GABAergic pathways, including α2 and α5, in GABA_A_ receptors can disrupt emotional processing and mood regulation in brain areas [15, 16, 65].

Human proteins are frequently used for molecular docking studies to support in vivo studies using laboratory animals, such as mice and chicks [40, 66, 67]. It is due to the ultimate target of this type of preclinical study to submit sufficient data to the clinical studies. This process helps us to find suitable drug candidates and their possible targets in humans [68]. In this study, we examined the docking score and interaction between PHY, DZP, and FLU with GABA_A_ receptor subunits (e.g., α1, α2, α3, α5, and γ2) of humans. The results suggest that PHY showed strong binding affinity (−7.2 kcal/mol) with GABA_A_ (α3 subunit). In general, a ligand with a binding affinity of more than −6.0 kcal/mol is considered to exhibit strong binding affinity, suggesting that it forms stable interactions with the target receptor [69, 70]. Additionally, PHY and DZP showed the same amino acid (ARG C: 156 and LEU C: 142) residues with GABA_A_ (α3 subunit). On the other hand, DZP and FLU showed lower binding affinity (−6.8 and − 6.9 kcal/mol, respectively) than PHY. Conversely, PHY and DZP showed similar binding affinity (−6.5 kcal/mol) with GABA_A_ (α2 subunit). In addition, DZP and PHY exhibited similar amino acid (HIS A: 129, PHE A: 127, TYR A: 187, and TYR A: 237) residues with the GABA_A_ (α2 subunit) receptor binding site. Moreover, DZP, FLU, and PHY showed promising binding interactions through several hydrophobic and HBs with the α1, α2, α3, α5, and γ2 subunits of the GABA_A_ receptor. Additionally, we investigated the docking scores and interactions of FLUX and PHY with the 5HT_1A_ receptor, finding that FLUX exhibited a higher binding affinity (−7.9 kcal/mol) than PHY (−6.7 kcal/mol), indicating stronger binding potential. FLUX's affinity is supported by its diverse interactions, including a halogen bond with ILE A: 410, a pi‐sigma interaction with PHE A: 494, and pi‐pi stacking with TRP A: 406, along with alkyl and pi‐alkyl interactions with ILE A: 410, ARG A: 487, ILE A: 491, and PHE A: 414, which likely stabilize it within the receptor's binding site. In contrast, PHY showed a slightly lower affinity due to fewer interaction types; while it formed pi‐sigma interactions with TRP A:406 and PHE A:414, along with alkyl bonds with ILE A:491 and ARG A:487. However, both PHY and FLUX exhibited interactions with the same critical amino acid residues within the 5HT1A receptor, specifically PHE A: 414, ILE A: 491, ARG A: 487, TRP A: 406, and PHE A: 494. These shared residues facilitated various stabilizing interactions, such as pi‐sigma, pi‐pi stacking, and alkyl or pi‐alkyl bonds, which are crucial in the overall stability and binding affinity of both ligands to the receptor. Furthermore, the formation of a complex between receptors and their target ligands relies on hydrogen‐bond interactions, which are critical for determining the specificity of ligand binding.

A conserved amino acid residue is an amino acid within a protein sequence that remains relatively unchanged across different species or within a family of related proteins over evolutionary time [71]. These residues are often critical for the structure or function of the protein, such as being part of the active site, involved in substrate binding, or maintaining the protein's overall stability and folding. Because these amino acids are essential for the protein's function, evolutionary pressures tend to conserve them, leading to minimal variation across different organisms or proteins [72, 73]. However, from the two pieces of literature, we found that six conserved arginines (arginine 34, 70, 77, 123, 135, and 224) are found in the human GABA_A_ receptor α5 subunit [74]. An alanine scan of the GABA_A_‐R α2 subunit revealed that mutations at many highly conserved residues (R269, L277, and K279) were detrimental to receptor function [75]. In addition, in our *in silico* study, we did not find any conserved amino acid interaction between the ligand and receptor compared to the literature. Furthermore, in the near future, researchers should work on this.

Drug‐*likeness* is a critical criterion for anticipating the ‘drug‐like’ features of a chemical molecule during the first phases of drug development and manufacturing process. It is measured through the drug's physicochemical characteristics, suggesting the drug's nature in connection to pharmacokinetics [66]. Lipinski's rule of five is a frequently used methodology for evaluating drug likeness and pharmacokinetics. Lipinski's rule of five states that a drug candidate should have a MW of 500 g/mol or less, no more than five HBD, no more than 10 HBA, and a lipophilicity (LogP_o/w_) of five or less [76]. In the current investigation, PHY demonstrated better pharmacokinetic properties and fulfilled Lipinski's role as a medication (Table 5). Additionally, PHY, DZP, and FLU each show favorable bioavailability and safety profiles. PHY showed high lipophilicity and low GI absorption. However, further studies should focus on optimizing the pharmacokinetic properties of PHY to enhance its therapeutic potential.

Toxicological screening is a crucial phase in the drug development process, which plays a role in selecting and ranking molecules with the best potential for safe and effective usage in humans. It also enhances the curative properties of existing compounds and reduces the likelihood of costly late‐stage losses [77]. Humans frequently experience many types of organ damage from prolonged chemical exposure, including neurotoxicity, genotoxicity, immunotoxicity, carcinogenicity, and developmental and reproductive toxicity [78]. In the current research, we use the web resources SwissADME, pkCSM, ADMETlab 3.0, and ProTox 3.0 for predicting the drug‐like qualities and ADMET features of PHY. The toxicity assessment of PHY, DZP, and FLU reveals marked differences, with PHY showing the highest tolerance (LD_50_ of 5000 mg/kg) and lowest toxicity classification (Class 5), followed by FLU (LD_50_ of 1300 mg/kg, Class 4), and DZP, which has the lowest tolerance (LD_50_ of 48 mg/kg, Class 2) and thus the highest acute toxicity. None of the compounds showed hepatotoxicity, carcinogenicity, immunotoxicity, or mutagenicity, suggesting overall favorable safety profiles. However, DZP's observed cytotoxicity could impact its safety in applications requiring high doses or extended use, presenting a potential limitation in its therapeutic window. On the other hand, PHY exhibits the highest tolerance with an LD50 of 5000 mg/kg, classified in toxicity Class 5, and shows no hepatotoxicity, carcinogenicity, immunotoxicity, or mutagenicity. Table 5 shows that all computed parameters for PHY (drug‐like characteristics and ADMET profile) stay within permissible ranges.

**FIGURE 4:**
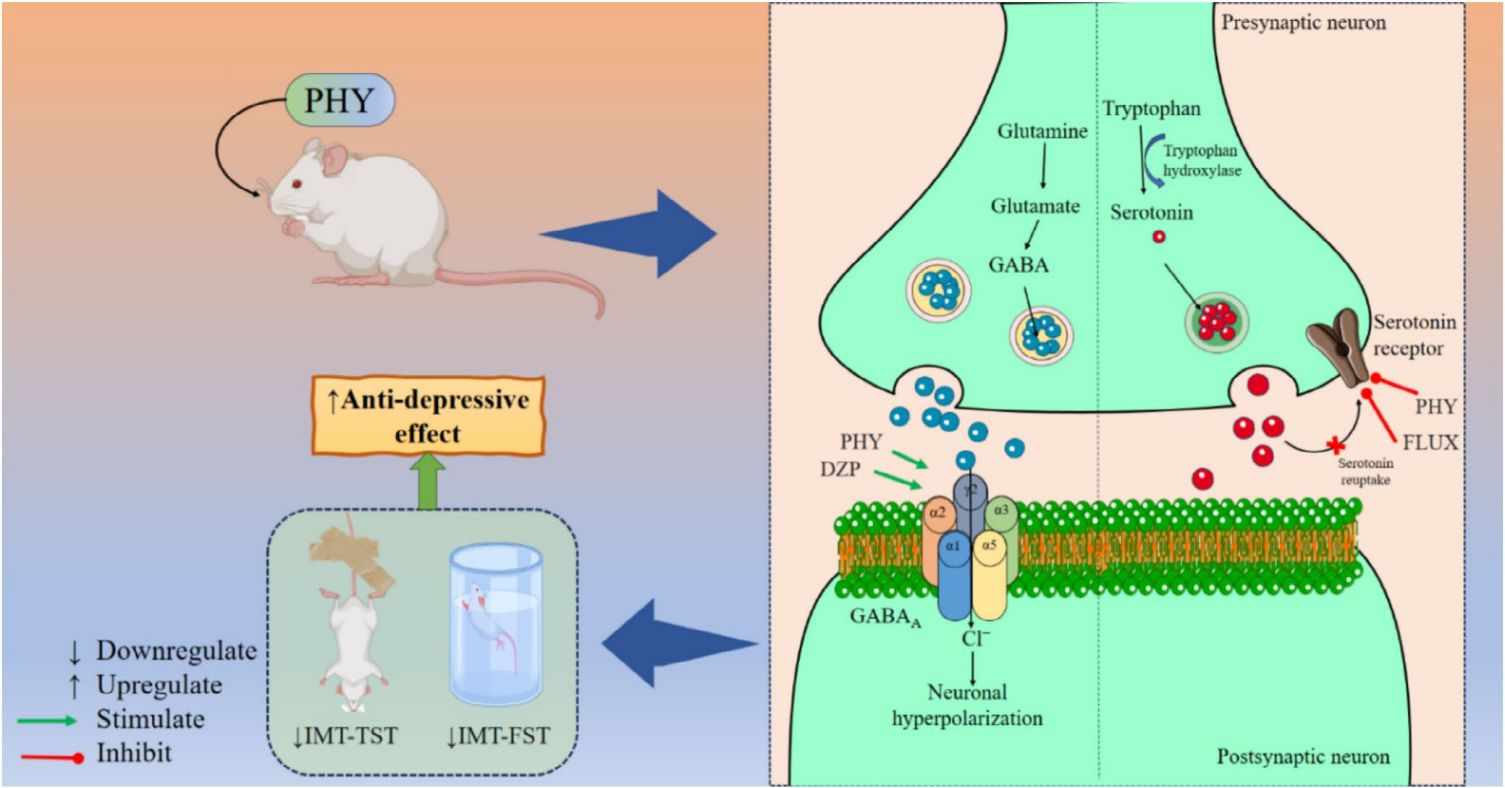
Possible antidepressant mechanism of phytol through GABAergic and serotonin pathways. [This figure illustrates the antidepressant mechanisms of PHY on the GABAergic and serotonergic systems. PHY, in combination with DZP, enhances GABA_A_ receptor activity, leading to increased Cl^−^ influx into postsynaptic neurons. This influx causes hyperpolarization, which decreases neuronal excitability and produces antidepressant effect. Meanwhile, PHY enhances serotonin synthesis by acting on tryptophan hydroxylase and inhibited serotonin receptor signaling on presynaptic neurons. Together, these actions elevate serotonin levels, contributing to mood regulation. The figure suggests that PHY's GABAergic modulation and serotonergic enhancement produce a combined antidepressant effect].

Taken together, our test result expressed that PHY showed concentration‐dependent GABA inhibitory power and a moderate antidepressant effect in experimental animals. Major depression is more prevalent among females than male animals. Female sex hormones make these differences, which are easy to study using animal studies like FST [79]. In this study, both FST and TST suggest that PHY augmented IMT values in female animals compared to the respective male groups, suggesting its sex‐dependent antidepressant effect on *Swiss* mice. The *in silico* study revealed the mechanisms or causes behind the antidepressant effect, potentially through the interaction between PHY and different AA residues of GABA_A_ and 5HT_1A_ receptors. The possible antidepressant mechanism of PHY in comparison to DZP, FLU, and FLUX is depicted in Figure 4.

## 5 Conclusion

PHY dose‐dependently and significantly (*p* < 0.05) reduced IMT values in TST and FST compared to the control and FLUX groups, suggesting its possible sedative‐like antidepressant effects in *Swiss* mice. However, it showed an augmented IMT value in female animals than in the respective male groups. PHY augmented IMT values with the standard GABAergic agonist drug DZP while reducing this parameter with the GABAergic antagonist drug FLU in animals. In vitro findings suggest that PHY and/or DZP concentration‐dependently and significantly (*p* < 0.05) inhibited GABA activity in test tubes while FLU and its combination reversed it. The *in silico* studies suggest that PHY exhibited the best binding affinities with the α2 and α3 subunits of the GABA_A_ and 5HT_1A_ receptors by −6.5, −7.2, and −6.7 kcal/mol, respectively. Taken together, PHY exerted a slight sex‐dependent sedative‐*like* antidepressant effect on *Swiss* mice. PHY may modulate the antidepressant effects of DZP, FLUX, and FLU through modulating IMT values in animals by interacting with the GABA_A_ receptor α2 and α3 subunits and 5HT_1A_ receptor. Further studies are highly appreciated to confirm the exact molecular mechanism for the antidepressant effects in animals and the safety profile of PHY.

## Author Contributions

**Md. Torequl Islam:** conceptualization, data curation, formal analysis, investigation, methodology, project administration, and writing – original draft. **Jannatul Ferdous, Md. Sakib Al Hasan, and Irfan Aamer Ansari:** software, and writing – original draft. **Md. Amirul Islam, Md. Shimul Bhuia, and Siddique Akber Ansari:** resources, and writing – review and editing. **Md. Saifuzzaman:** project administration, supervision, validation, and visualization.

## Conflicts of Interest

The authors declare no conflicts of interest.

## Data Availability

Data will be made available upon request.
